# Kupffer Cell–Derived Exosomal S100A8/A9 Promotes Inflammasome–Dependent Pyroptotic Lung Injury in Acute Pancreatitis

**DOI:** 10.1016/j.jcmgh.2026.101810

**Published:** 2026-05-12

**Authors:** Wen-Qi He, Yi Liu, Ying-Rui Tang, Rui Wang, Chang-Ping Chen, Xiao-Qin Lv, Yuan-Fei Sha, Jian-Dong Ren

**Affiliations:** 1 Department of Pharmacy, Sichuan Provincial People’s Hospital, University of Electronic Science and Technology of China, Chengdu, China; 2 Innovation Center of Advanced Pharmaceutical and Artificial Intelligence, School of Medicine, University of Electronic Science and Technology of China, Chengdu, China; 3 Department of Anesthesiology, Changhai Hospital, Naval Medical University, Shanghai, China; 4 Department of Medical Technology, Xichang Medical College, Xichang, China

**Keywords:** Acute Pancreatitis, Heat Shock Protein 70, Kupffer Cell, NLRP3 Inflammasome, Pyroptosis

## Abstract

**Background & Aims:**

Acute pancreatitis frequently progresses to systemic inflammation and acute lung injury, but the circulating mediators that couple pancreatic inflammation to remote organ damage remain poorly characterized. Following our finding that plasma exosomes induce acute pancreatitis–associated lung injury by triggering NOD-like receptor protein 3–dependent pyroptotic death in alveolar macrophages, this study aims to identify exosome-encapsulated S100A8/A9 derived from Kupffer cells as a critical propagator of this inflammatory cascade.

**Methods:**

Levels of S100A8/A9 in plasma-derived exosomes from mice with acute pancreatitis were quantified and correlated with pulmonary cytokine profiles. Plasma exosomes were characterized and administered to alveolar macrophages and healthy mice, followed by evaluation of their capacity to induce pyroptosis and lung injury. The specific role of S100A8/A9 was tested using genetic knockout and/or pharmacologic inhibition. Furthermore, Kupffer cells were investigated as a putative cellular source of these exosomes by utilizing a combination of in vitro stimulation and in vivo depletion models. Finally, the role of S100A8/A9 in perturbing the lysosomal pathway was mechanistically dissected to reveal the pathway underlying NOD-like receptor protein 3 inflammasome activation and pyroptosis.

**Results:**

Elevated plasma S100A8/A9 levels paralleled pulmonary cytokines increases in mice with acute pancreatitis, and the circulating S100A8/A9 was predominantly carried by exosomes. Rapidly internalized by alveolar macrophages, acute pancreatitis-derived exosomes delivered S100A8/A9 to induce pyroptosis. Adoptive transfer of acute pancreatitis–derived exosomes into healthy mice reproduced lung injury, whereas exosomes lacking S100A9 or from Kupffer cell–depleted acute pancreatitis mice caused markedly less pathologic alterations. Mechanistically, exosomal S100A8/A9 activated the NOD-like receptor protein 3 inflammasome and executed pyroptosis through a pathway via suppression of heat shock protein 70–dependent acid sphingomyelinase, leading to sphingomyelin accumulation and lysosomal membrane permeabilization.

**Conclusions:**

These findings uncover a pancreas-liver-lung inflammatory axis in which Kupffer cell–derived exosomal S100A8/A9 drives systemic inflammation in acute pancreatitis, representing a tractable therapeutic target to prevent extrapancreatic organ injury.

## Graphical abstract

**Figure:**
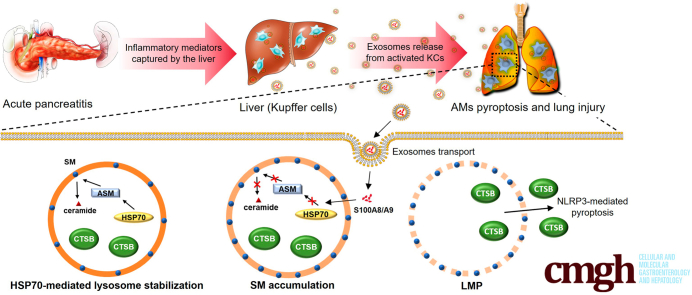

**Summary**

This study identifies a pancreas–liver–lung inflammatory axis in acute pancreatitis in which Kupffer cell–derived exosomal S100A8/A9 promotes alveolar macrophage pyroptosis through disruption of heat shock protein 70–dependent lysosomal homeostasis. These findings highlight S100A8/A9 as a potential therapeutic target for acute pancreatitis–associated organ injury.

**What You Need to Know**

**Background**

Acute pancreatitis is commonly complicated by lung injury that drives disease severity and mortality, yet the interorgan signaling and intracellular pathways governing this process remain incompletely characterized.

**Impact**

Kupffer cell–derived exosomal S100A8/A9 drives NOD-like receptor protein 3–dependent pyroptosis in alveolar macrophages by impairing heat shock protein 70–mediated lysosomal membrane stabilization, establishing a pancreas–liver–lung inflammatory axis during acute pancreatitis.

**Future Directions**

Exosomal S100A8/A9 may serve as a mechanistic driver and potential therapeutic target for acute pancreatitis–associated extrapancreatic organ injury, providing a framework for intervention strategies that require further clinical validation.

Acute pancreatitis (AP) is a common emergency condition characterized by an abrupt inflammatory response initiated by pancreatic injury through diverse pathogenic mechanisms. Approximately 20% of patients develop severe AP, which is associated with mortality rates as high as 30% to 40%, representing a substantial clinical challenge.^1^ A hallmark of AP progression is the rapid dissemination of inflammatory mediators from the pancreas to distant organs, leading to a systemic inflammatory response that critically determines disease severity and outcome.^2^ Among extrapancreatic complications, acute lung injury (ALI) is the most frequent and lethal, accounting for up to 60% of AP-related deaths.^3^ To date, however, the mechanisms by which pancreatic inflammation propagates to the lung and culminates in ALI remain incompletely defined.

Recent advances have highlighted the importance of intercellular communication in orchestrating systemic inflammation during AP. In particular, exosomes, nanosized extracellular vesicles released by diverse cell types, have emerged as potent conveyors of inflammatory signals through the transfer of bioactive cargo, thereby shaping immune responses and inflammatory cascades.^4^ Accumulating evidence indicates that circulating exosomes are not merely biomarkers, but active pathogenic mediators driving the progression of AP toward severe disease and multiorgan dysfunction.^5^, ^6^, ^7^, ^8^, ^9^ Consistent with this notion, our previous work demonstrated that circulating exosomes induce NOD-like receptor protein 3 (NLRP3) inflammasome–dependent pyroptosis in alveolar macrophages (AMs), thereby directly contributing to AP-associated pulmonary injury.^10^ Although our finding has provided deeper insights into the pathogenesis of AP-associated ALI, the precise molecular mechanisms particularly concerning the cellular or tissue origin of pathogenic circulating exosomes and the specific exosomal components responsible for inflammasome activation remain to be fully clarified.

The proinflammatory alarmin S100A8/S100A9 (S100A8/A9) heterodimer is abundantly expressed in early infiltrating phagocytes and rapidly released upon their activation, while remaining minimally expressed or undetectable in healthy tissues.^11^ Previous studies have reported marked enrichment of S100A8/A9 in circulating exosomes derived from AP models.^6^^,^^8^ However, the functional contribution of exosome-associated S100A8/A9 to systemic inflammation and distant organ injury in AP has not been defined. Given that S100A8/A9 has been shown to activate the NLRP3 inflammasome and drive tissue injury in sepsis-associated lung injury, acute kidney injury, myelodysplastic syndromes, and acute coronary syndromes,^12^, ^13^, ^14^, ^15^ we hypothesized that exosome-delivered S100A8/A9 acts as a previously unrecognized effector that licenses AMs pyroptosis and promotes AP-associated lung injury.

Building on this hypothesis, a critical unresolved issue concerns the cellular or tissue origin of S100A8/A9–enriched circulating exosomes during AP. Because AP-associated ALI represents a prototypical form of remote organ injury driven by systemic inflammatory amplification, the source of pathogenic circulating exosomes is unlikely to be confined to the pancreas. Compelling evidence indicates that pancreas-derived inflammatory mediators are intercepted by the liver via the portal circulation, where they activate Kupffer cells (KCs) to release secondary inflammatory mediators, thereby amplifying systemic inflammation. Notably, depletion of KCs alleviates pulmonary injury without ameliorating pancreatic pathology, highlighting the liver’s central role in shaping distal organ damage during AP.^16^, ^17^, ^18^, ^19^, ^20^ Accordingly, the liver has been recognized as an “inflammatory amplifier” in AP progression. In further support of this notion, several proteins highly enriched in AP plasma exosomes, including mannose-binding protein C, haptoglobin, and apolipoprotein C-I, are either liver-specific or predominantly expressed in hepatic tissue, implicating the liver as a major source of circulating exosomes.^6^ Thus, these observations led us to propose that hepatic KCs represent a dominant cellular source of pathogenic circulating exosomes that establish a pancreas–liver–lung inflammatory axis in AP. In this study, we employed complementary in vitro and in vivo approaches to determine whether KC-derived exosomes are the principal instigators of AMs pyroptosis and AP-associated lung injury, with a particular focus on how exosomal S100A8/A9 mechanistically licenses NLRP3 inflammasome activation and pyroptosis. By delineating this previously unappreciated interorgan inflammatory circuit, our work provides mechanistic insight into AP-associated lung injury and identifies novel therapeutic targets for preventing extrapancreatic organ dysfunction in AP.

## Results

### Exosomal S100A8/A9 as the Dominant Driver of Elevated Plasma Alarmin and Pulmonary Inflammation in Experimental Acute Pancreatitis

**Figure 1:**
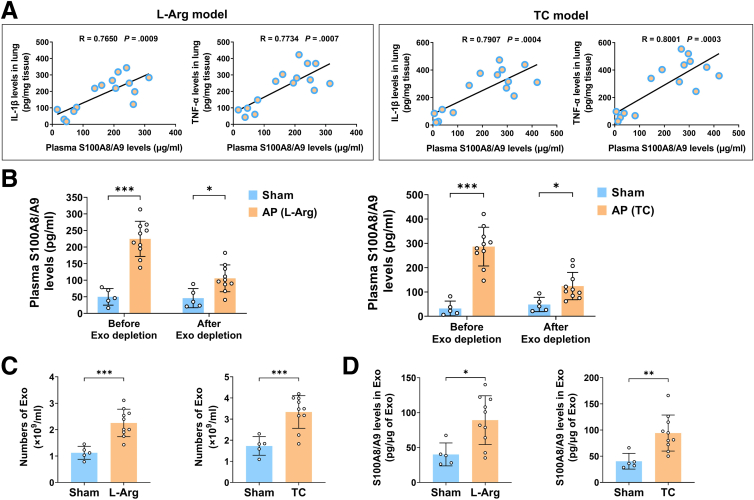
**Exosomal S100A8/A9 was the dominant driver of the plasma alarmin surge and pulmonary inflammation during AP.** (*A*) Linear regression analysis between pulmonary proinflammatory cytokine levels and plasma S100A8/A9 levels from AP mice challenged with L-Arg or TC. (*B*) Plasma levels of S100A8/A9 before and after exosome depletion. (*C*) The amount of exosomes in plasma. (*D*) The levels of S100A8/A9 in exosomes. ∗*P* < .05, ∗∗*P* < .01, ∗∗∗*P* < .001.

In AP murine models induced by L-arginine (L-Arg) or taurocholate (TC), plasma S100A8/A9 levels were assessed for correlation with pulmonary proinflammatory cytokine levels. As illustrated in Figure 1*A*, significant positive correlations were observed between interleukin-1 beta (IL-1β)/tumor necrosis factor α levels in lung tissue and circulating level of S100A8/A9 across both AP models. To determine the contribution of exosome-loaded S100A8/A9 to plasma level of S100A8/A9, exosomes were depleted from plasma using polyethylene glycol (PEG). Markedly elevated plasma S100A8/A9 levels in both AP models were observed compared with the sham-operated controls (*P* < .001). Depletion of exosomes reduced, yet did not abolish, the increment (.01 < *P* < .05) (Figure 1*B*), indicating that the significant portion of the S100A8/A9 in plasma is exosome-associated. Consistent with this interpretation, AP mice exhibited a parallel rise in circulating exosome number (Figure 1*C*) and a substantial enrichment of S100A8/A9 within these vesicles (Figure 1*D*). Collectively, these data establish exosome-encapsulated S100A8/A9 as the dominant driver of the plasma alarmin surge and pulmonary inflammation during AP.

### Plasma-Derived Exosomes From Acute Pancreatitis Mice Delivered S100A8/A9 Into Alveolar Macrophages

**Figure 2:**
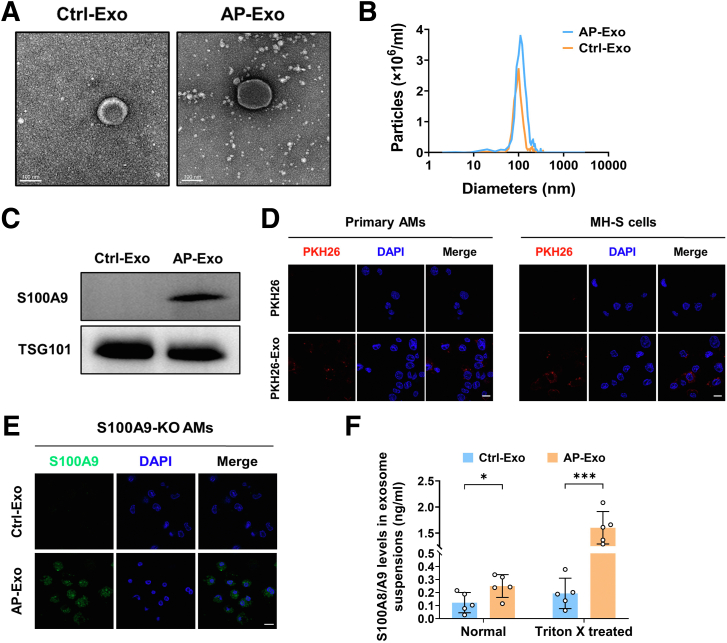
**Plasma-derived exosomes from AP mice delivered S100A8/A9 into AMs.** (*A*) Exosomes collected from plasma were resuspended in PBS and observed by transmission electron microscopy (scale bar, 100 nm). (*B*) Size distribution of exosomes collected from plasma. (*C*) The protein levels of exosomal S100A9 and surface marker (TSG101) were analyzed by Western blotting. (*D*) The PKH26-Exo taken up by primary AMs or MH-S cells are visible inside the cells (Scale bar, 20 μm). (*E*) S100A9-KO primary AMs were incubated with exosomes, and cellular S100A9 was visualized by immunofluorescence analysis. (*F*) The levels of S100A8/A9 in exosome suspensions were detected by ELISA before and after the treatment of Triton X-100. ∗*P* < .05, ∗∗∗*P* < .001.

Exosomes isolated from mouse plasma displayed the characteristic cup-shaped morphology and bilayer membrane architecture of nanoscale extracellular vesicles (Figure 2*A*). Nanoparticle tracking analysis revealed a narrow size distribution (60–150 nm) that was indistinguishable between AP-derived exosomes (AP-Exo) and control exosomes (Ctrl-Exo), but the particle concentration was significantly higher in the AP group (Figure 2*B*). Because S100A9 monomers induce pyroptotic cell death to a similar extent as the S100A8/A9 heterodimer, detection of S100A9 alone in plasma exosomes reliably reflects the presence and propyroptotic activity of the heterodimer. Immunoblotting demonstrated pronounced loading of S100A9 in AP-Exo, whereas it was virtually absent in Ctrl-Exo (Figure 2*C*). Cellular fluorescence imaging of PKH26-labeled vesicles showed rapid internalization into primary AMs and MH-S cells (Figure 2*D*), an event that was accompanied by a measurable increase in intracellular S100A9 abundance (Figure 2*E*). To determine whether S100A8/A9 is encapsulated within exosomes or associated with their surface, we measured S100A8/A9 levels in exosome suspensions by enzyme-linked immunosorbent assay (ELISA) before and after Triton X-100 treatment, which disrupts exosomal membrane integrity. As shown in Figure 2*F*, S100A8/A9 levels in intact AP-Exo were marginally higher than those in Ctrl-Exo (*P* = .0408). In contrast, following Triton X-100-mediated membrane disruption, S100A8/A9 levels in AP-Exo were markedly elevated compared with Ctrl-Exo, achieving a highly significant difference (*P* < .001). These results indicate that S100A8/A9 is predominantly encapsulated within exosomes, as the intact membrane structure limits antibody accessibility in the assay.

### Exosome-Encapsulated S100A8/A9 Mediated Acute Pancreatitis–Exosome-Induced Alveolar Macrophage Pyroptosis

**Figure 3:**
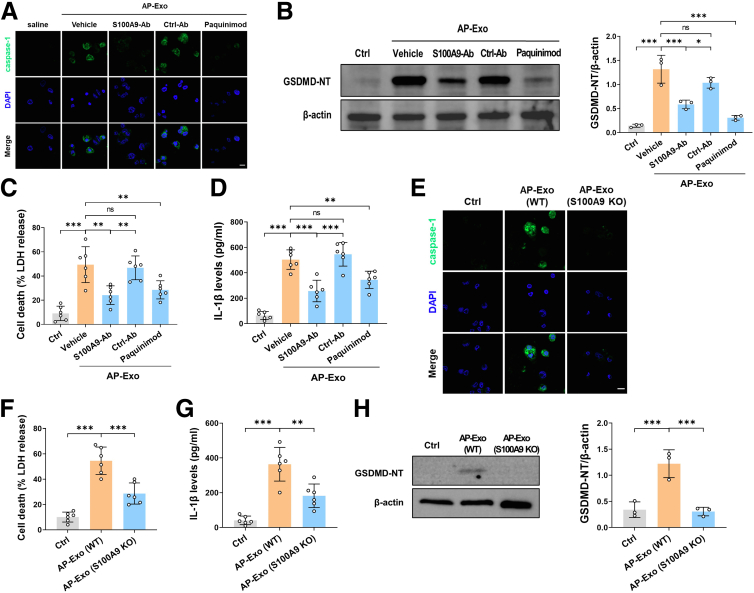
**Exosome-encapsulated S100A8/A9 mediated AP-exosome–induced AMs pyroptosis.** (*A*) Intracellular activated caspase-1 was visualized under confocal microscope after AP plasma exosome (AP-Exo)-treated MH-S cells were stained with FAM-FLICA reagent (Scale bar, 20 μm). (*B*) The intracellular protein expression of GSDMD-NT fragment was determined by Western blotting. (*C*) The release of LDH into the culture medium was measured by colorimetric assay to quantify cell death. (*D*) The concentration of IL-1β in culture supernatants was measured by ELISA. (*E*) Intracellular activated caspase-1 was visualized with FAM-FLICA staining after cells were treated by AP-Exo (Scale bar, 20 μm). (*F*) The release of LDH into the culture medium was measured by colorimetric assay to quantify cell death. (*G*) The concentration of IL-1β in culture supernatants was measured by ELISA. (*H*) The intracellular protein expression of GSDMD-NT fragment was determined by Western blotting. ∗∗*P* < .01, ∗∗∗*P* < .001, ns: not significant, *P* > .05.

To delineate the contribution of exosome-delivered S100A8/A9 to AMs pyroptosis, Lipopolysaccharide (LPS)-primed MH-S cells were exposed to AP plasma exosomes in the presence of a neutralizing anti-S100A9 antibody or the small-molecule inhibitor Paquinimod. Although AP-Exo drove significant caspase-1 activation and the generation of the gasdermin-D (GSDMD) pore-forming fragment, along with robust release of lactate dehydrogenase (LDH) and IL-1β, each response was substantially blunted by S100A9 blockade, whereas an isotype control antibody failed to exhibit such effects (Figure 3*A–D*). To corroborate these pharmacologic findings with a genetic approach, AP was induced with L-Arg in S100A9 knockout (KO) mice to generate AP-Exo that is inherently devoid of S100A9. Compared with wild-type AP-Exo, S100A9-deficient AP-Exo exhibited a severely curtailed capacity to trigger caspase-1 activation, LDH release, IL-1β secretion, and GSDMD cleavage in LPS-primed MH-S cells (Figure 3*E–H*). Overall, these data establish S100A8/A9 as an essential pyroptotic cargo within AP plasma exosomes.

### Exosomal S100A8/A9 From Acute Pancreatitis–Conditioned Plasma Induced Alveolar Macrophage Pyroptosis and Lung Injury in Healthy Mice

**Figure 4:**
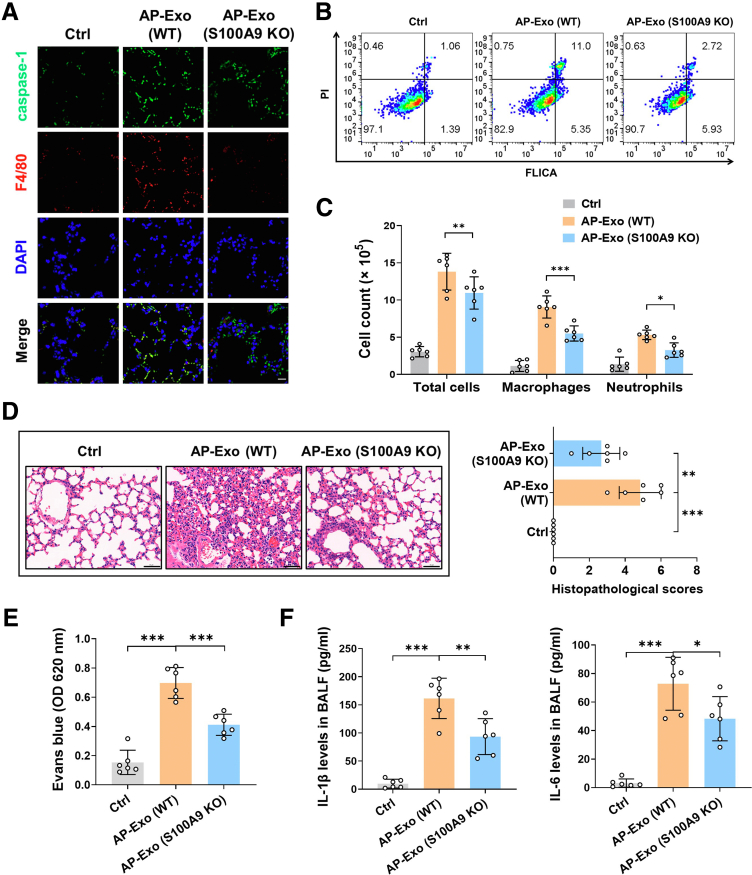
**Exosomal S100A8/A9 from AP plasma caused AM pyroptosis and lung injury in healthy mice.** (*A*) Immunofluorescence staining of activated caspase-1 and F4/80 in lung sections of mice after intravenous injection of AP-Exo (Scale bar, 50 μm). (*B*) Pyroptosis of AMs collected from mice after intravenous administration of AP-Exo was determined by FAM-FLICA/PI double-staining using flow cytometry. (*C*) Cell counts in BALF of mice after intravenous injection of AP-Exo. (*D*) Histopathologic evaluation was performed under light microscope after the lung sections were stained with H&E (Scale bar, 50 μm) and the histopathologic scores were calculated. (*E*) Pulmonary vascular permeability was assessed by Evans blue dye extravasation. (*F*) The levels of cytokines (IL-1β and IL-6) in BALF were measured by ELISA. ∗*P* < .05, ∗∗*P* < .01, ∗∗∗*P* < .001. H&E, hematoxylin and eosin; PI, propidium iodide.

We next asked whether exosome-embedded S100A8/A9 is sufficient to ignite pulmonary inflammation in healthy mice. Intravenous administration of wild-type AP-Exo evoked robust caspase-1 activation that colocalized with the macrophage marker F4/80 in lung sections. In contrast, mice given AP-Exo isolated from S100A9-deficient donors exhibited a marked reduction in caspase-1 activation, although residual signal remained detectable (Figure 4*A*). Flow-cytometric quantification of pyroptotic AMs corroborated this dependency (Figure 4*B*). It was found that exosomal S100A9 was also required for the ensuing inflammatory cell recruitment. Exposure to AP-Exo resulted in a substantial rise in lung-infiltrating macrophages and neutrophils, whereas exosomes derived from S100A9-deficient mice displayed a markedly reduced capacity to recruit these cells, with macrophage accumulation showing the most prominent decline (*P* < .001) (Figure 4*C*). Functional consequences were evaluated by histopathology and alveolar–capillary permeability. Mice receiving intact AP-Exo exhibited thickened alveolar septa and interstitial edema, accompanied by significant increase in Evans-blue leakage. These abnormalities were largely mitigated when S100A9-null exosomes were administered (Figure 4*D* and *E*). Moreover, the activity of AP-Exo to elevate IL-1β and interleukin-6 (IL-6) levels in bronchoalveolar lavage fluid (BALF) of healthy mice was markedly attenuated when S100A9 was absent from the vesicles (Figure 4*F*). These data confirm exosome-encapsulated S100A8/A9 as a critical instigator of AM pyroptosis and consequent lung injury in vivo.

### The Exosomes Triggering Alveolar Macrophage Pyroptosis During Acute Pancreatitis Were Generated From Hepatic Kupffer Cells

**Figure 5:**
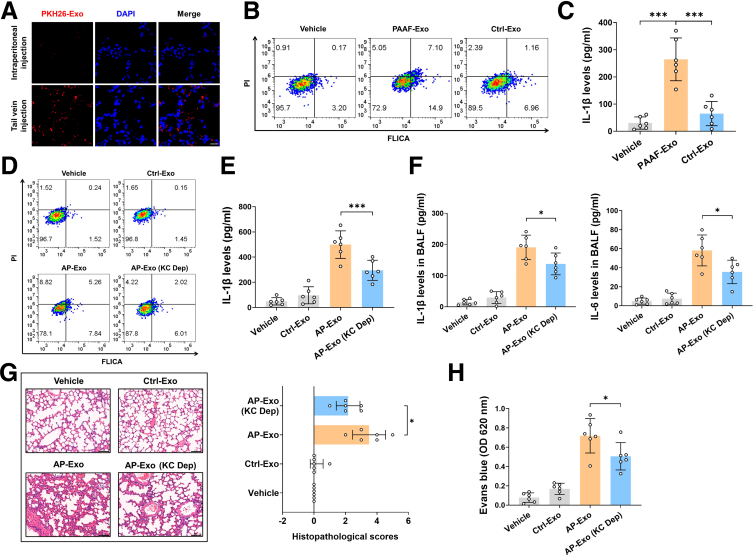
**The exosomes triggering AMs pyroptosis during AP were generated from hepatic KCs.** (*A*) Representative images of lung sections collected from mice intravenously or intraperitoneally injected with PKH26-Exo under confocal microscope (Scale bar, 50 μm). (*B*) Pyroptosis of AMs stimulated by PAAF-Exo was determined by FAM-FLICA/PI double-staining using flow cytometry. (*C*) The level of IL-1β in culture supernatants was measured by ELISA. (*D*) Pyroptosis of AMs was determined by FAM-FLICA/PI double-staining using flow cytometry after stimulation with plasma exosomes collected from control or KC-depleted AP mice. (*E*) IL-1β release by AMs in response to AP-Exo was measured by ELISA. (*F*) Cytokine (IL-1β and IL-6) levels in BALF of mice after intravenous injection of AP-Exo. (*G*) Histopathologic evaluation was performed under light microscope after the lung sections were stained with H&E (Scale bar, 50 μm), and the histopathologic scores were calculated. (*H*) Pulmonary vascular permeability was assessed by Evans blue dye extravasation. ∗*P* < .05, ∗∗∗*P* < .001. H&E, hematoxylin and eosin; PI, propidium iodide.

Having established exosomal S100A8/A9 as the principal culprit driving AP-associated lung inflammation, we next sought to define its tissue origin. Tail-vein injection of AP-Exo delivered considerably more vesicles to the lung than did intraperitoneal administration, implying that exosomes absorbed from the peritoneal cavity are largely sequestered by the liver (Figure 5*A*). We therefore reasoned that KCs, the liver resident macrophages, pick up pancreas-derived inflammatory cues and then release the lung-targeting exosomes. To validate the speculation, KCs were stimulated with pancreatitis-associated ascitic fluid (PAAF) to mimic post-AP hepatic exposure. Consistent with this hypothesis, exosomes released by PAAF-stimulated KCs (PAAF-Exo) induced robust caspase-1 activation and IL-1β release in LPS-primed AMs (Figure 5*B* and *C*). Once KCs were depleted with clodronate liposomes prior to L-Arg–induced AP, plasma exosomes exhibited a markedly diminished capacity to elicit AMs pyroptosis in vitro (Figure 5*D* and *E*). In vivo findings mirrored the in vitro data, with exosomes from KC-depleted AP mice provoking noticeably less cytokines release than AP-Exo from KC-intact mice (Figure 5*F*). Consequently, pulmonary histopathology and alveolar–capillary leak caused by AP-Exo injection were significantly attenuated when vesicles were isolated from KC-depleted animals (Figure 5*G* and *H*). Together, these results demonstrate that pancreas-derived inflammatory cues recruit KCs as the dominant hepatic source of S100A8/A9-rich exosomes that, once released into the circulation, precipitate AM pyroptosis and consequent lung injury.

### Exosomal S100A8/A9 Derived From Kupffer Cells Caused Alveolar Macrophage Pyroptosis and Acute Pancreatitis–Related Lung Injury

**Figure 6:**
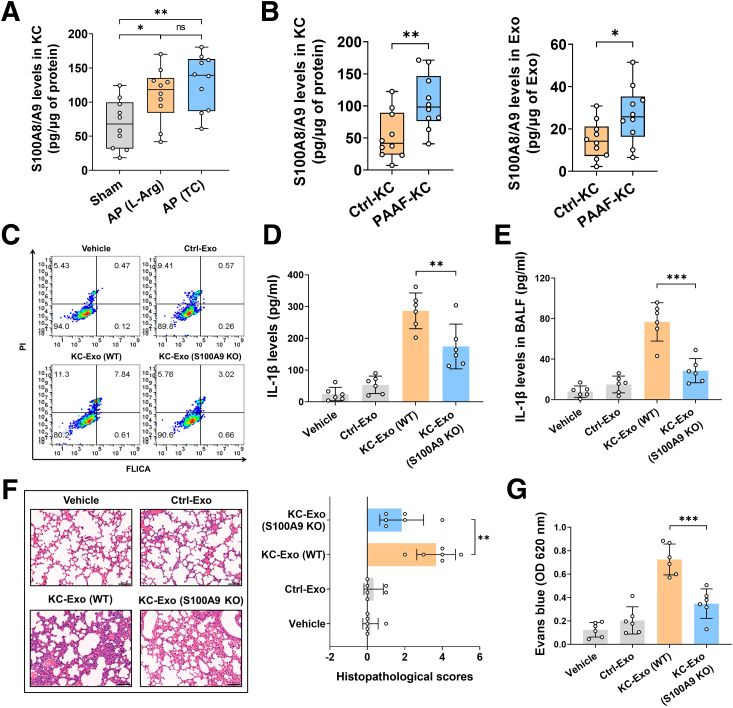
**Exosomal S100A8/A9 derived from KCs caused AMs pyroptosis and AP-related lung injury.** (*A*) Protein expression level of S100A8/A9 in KCs collected from AP mice induced by L-Arg or TC. (*B*) Protein levels of S100A8/A9 in KCs and KC-secreted exosomes in response to PAAF. (*C*) Pyroptosis of AMs was determined by FAM-FLICA/PI double-staining using flow cytometry after stimulated with KC-derived exosomes under various conditions. (*D*) IL-1β release by AMs in response to KC-derived exosomes. (*E*) IL-1β level in BALF of mice after intravenous injection of KC-derived exosomes under various conditions. (*F*) Histopathologic evaluation was performed under light microscope after the lung sections were stained with H&E (scale bar, 50 μm), and the histopathologic scores were calculated. (*G*) Pulmonary vascular permeability was assessed by Evans blue dye extravasation. ∗*P* < .05, ∗∗*P* < .01, ∗∗∗*P* < .001, ns: not significant, *P* > .05. H&E, hematoxylin and eosin; PI, propidium iodide.

S100A8/A9 expression was significantly up-regulated in hepatic KCs from both L-Arg– and TC-induced AP mice (Figure 6*A*). Moreover, PAAF challenge in vitro also increased S100A8/A9 level in KCs and further enriched S100A8/A9 within KC-derived exosomes (Figure 6*B*). To test functionality, we isolated KCs from S100A9-KO mice, stimulated them with PAAF, and harvested the released exosomes. Compared with exosomes released by unstimulated KCs (Ctrl-Exos), exosomes secreted following PAAF challenge (KC-Exo-WT) provoked robust AMs pyroptosis and a parallel surge in IL-1β production, whereas this response was markedly attenuated when the parental KCs lacked S100A9 (Figure 6*C* and *D*). The pathogenic impact of these vesicles was next examined in vivo. Intravenous delivery of KC-Exo-WT into healthy mice raised IL-1β level in BALF, increased alveolar–capillary permeability and produced overt histopathologic changes that were significantly attenuated when exosomes originated from S100A9-null KCs (Figure 6*E–G*). Collectively, the data establish S100A8/A9 within KC-derived exosomes as the principal effector responsible for triggering AM pyroptosis and resultant lung injury during AP.

### S100A8/A9 Induced NOD-Like Receptor Protein 3 Inflammasome Activation and Pyroptosis by Triggering Lysosomal Destabilization

**Figure 7:**
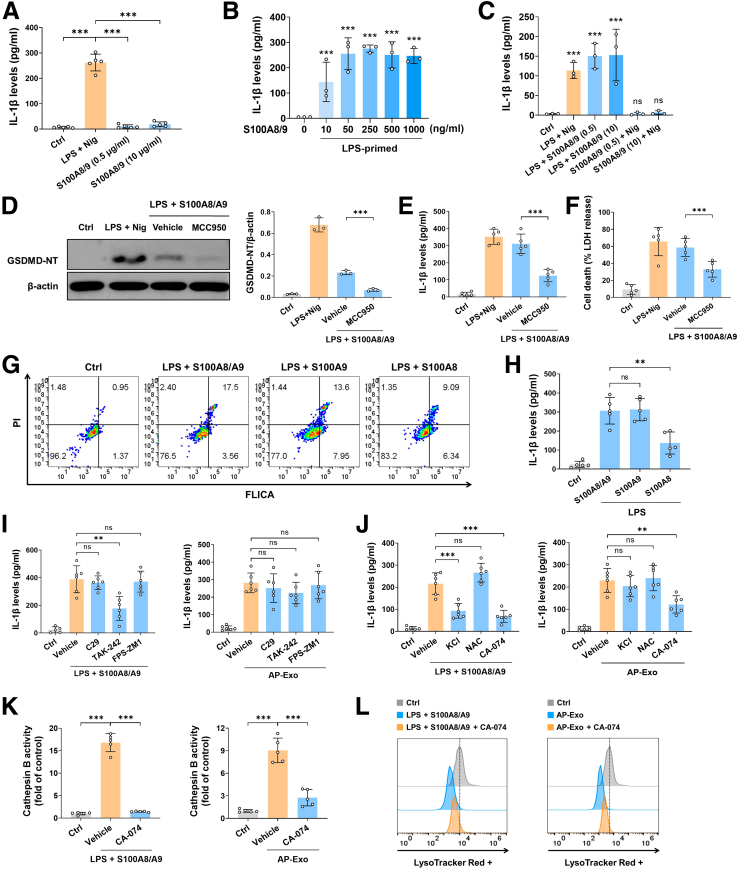
**S100A8/A9 induced NLRP3 inflammasome activation and pyroptosis by triggering lysosomal destabilization.** (*A*) Unprimed THP-1 cells were treated with only S100A8/A9 heterodimer or LPS-primed THP-1 cells were challenged with nigericin. IL-1β production was measured by ELISA. (*B*) IL-1β release by LPS-primed THP-1 cells after stimulated with various concentrations of S100A8/A9. (*C*) IL-1β production in S100A8/A9 (0.5, 10 μg/mL) primed THP-1 cells followed by nigericin stimulation or in LPS-primed THP-1 cells followed by the treatment of S100A8/A9 (0.5, 10 μg/mL). (*D*) The intracellular protein expression of GSDMD-NT fragment in LPS-primed cells after the treatment of nigericin or S100A8/A9. (*E*) IL-1β release by LPS-primed THP-1 cells after the treatment of nigericin or S100A8/A9. (*F*) The release of LDH into the culture medium by LPS-primed cells after the treatment of nigericin or S100A8/A9. (*G*) Pyroptosis of LPS-primed MH-S cells determined by FAM-FLICA/PI double-staining using flow cytometry after stimulated with S100A8/A9 heterodimer or monomer. (*H*) IL-1β release by LPS-primed MH-S cells after stimulated with S100A8/A9 heterodimer or monomer. (*I*) Production of IL-1β in LPS + S100A8/A9– or AP-Exo–stimulated cells in the presence of various receptor antagonists. (*J*) Production of IL-1β in LPS + S100A8/A9– or AP-Exo–stimulated cells in the presence of various pathway-specific inhibitors. (*K*) Cathepsin B activity in LPS + S100A8/A9– or AP-Exo–stimulated cells measured by a fluorescence-based assay. (*L*) The lysosomal integrity of cells analyzed by flow cytometry after stained with LysoTracker Red. ∗∗*P* < .01, ∗∗∗*P* < .001, ns: not significant, *P* > .05. PI, propidium iodide.

It is known that canonical NLRP3 inflammasome activation is triggered only when a priming step (Signal 1) is followed by an inflammasome-assembly stimulus (Signal 2). S100A8/A9 was reported previously to possess the capacity to induce the NLRP3 inflammasome activation. To map S100A8/A9 within the 2-step inflammasome circuit, we exposed unprimed THP-1 cells to the S100A8/A9 heterodimer and found it alone failed to evoke mature IL-1β secretion (Figure 7*A*), indicating it does not deliver Signal 1. However, when added to LPS-primed cells, it triggered robust IL-1β secretion that saturated at intermediate doses (Figure 7*B*). Reciprocally, cells primed only with S100A8/A9 remained unresponsive to nigericin (Figure 7*C*), indicating that the heterodimer supplies Signal 2 but not Signal 1 for the activation of NLRP3 inflammasome. Consistently, S100A8/A9 together with LPS augmented the generation of the GSDMD pore-forming fragment, IL-1β release, and LDH leakage, all of which were blocked by the specific NLRP3 inhibitor MCC950 (Figure 7*D–F*). Thus, S100A8/A9 acts exclusively as a Signal 2 that licenses NLRP3 inflammasome activation and pyroptosis execution. Furthermore, S100A9 monomer exhibited comparable potency to the S100A8/A9 heterodimer in triggering AMs pyroptosis, and both elicited stronger responses than S100A8 monomer, as determined by caspase-1 activity and IL-1β production (Figure 7*G* and *H*).

S100A8/A9-driven inflammation is thought to engage Toll-like receptor (TLR) 2, TLR4, and/or receptor for advanced glycation end-products (RAGE). In LPS-primed macrophages, the TLR4 antagonist TAK-242 abolished alarmin-induced IL-1β release, whereas TLR2 inhibitor C29 or RAGE blocker FPS-ZM1 failed to exert a similar effect (Figure 7*I*). Remarkably, none of these antagonists curbed IL-1β secretion evoked by AP-Exo, implying that vesicle delivery bypasses conventional surface receptors. Turning to intracellular checkpoints, it was found that cathepsin B blockade with CA-074Me or inhibition of K^+^ efflux with KCl each blunted S100A8/A9-induced IL-1β secretion, whereas reactive oxygen species (ROS) scavenging with N-acetylcysteine (NAC) was ineffective. However, AP-Exo–stimulated release of IL-1β remained sensitive exclusively to cathepsin B blockade (Figure 7*J*). Consistently, both S100A8/A9 and AP-Exo provoked cathepsin B activation and lysosomal destabilization in LPS-primed macrophages, events reversed by CA-074Me (Figure 7*K* and *L*). Thus, lysosomal cathepsin B release constitutes the universal trigger for NLRP3-driven pyroptosis elicited by free or exosome-delivered S100A8/A9.

### Disruption of the Heat Shock Protein 70–Acid Sphingomyelinase Axis Licensed S100A8/A9-Driven NLRP3 Inflammasome Activation and Pyroptosis

**Figure 8:**
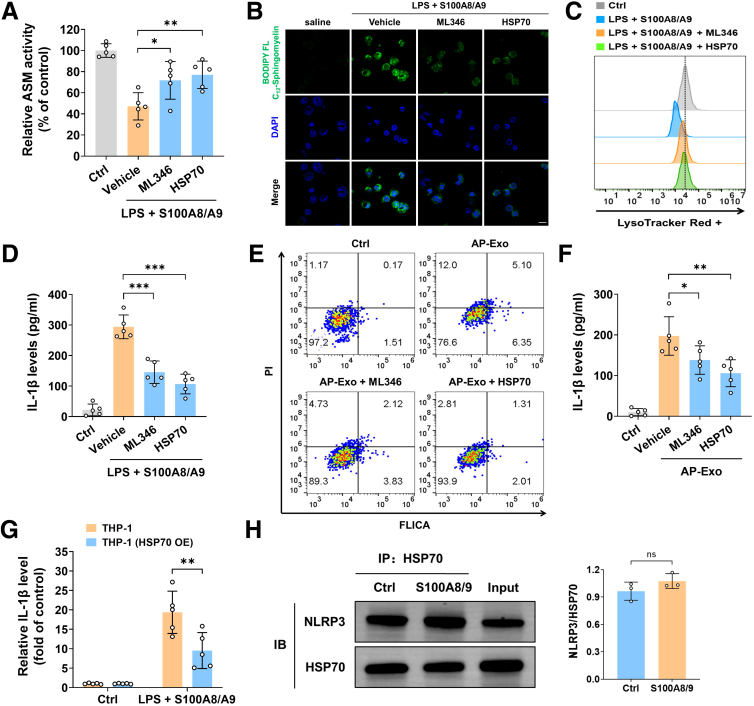
**Disruption of the HSP70-ASM axis licensed S100A8/A9-driven NLRP3 inflammasome activation and pyroptosis.** (*A*) ASM activity in LPS + S100A8/A9 treated cells in the presence of HSP70 or ML346 was determined using an ASM-specific fluorogenic substrate. (*B*) The sphingomyelin accumulation in LPS + S100A8/A9–treated cells in the presence of HSP70 or ML346 was visualized by BODIPY FL C_12_-Sphingomyelin staining under confocal microscope (scale bar, 20 μm). (*C*) The lysosomal integrity of cells analyzed by flow cytometry after being stained with LysoTracker Red. (*D*) Production of IL-1β in LPS + S100A8/A9–stimulated cells in the presence of HSP70 or ML346. (*E*) Pyroptosis of AMs was determined by FAM-FLICA/PI double-staining using flow cytometry after the stimulation with AP-Exo in the presence of HSP70 or ML346. (*F*) IL-1β release by AP-Exo–stimulated AMs in the presence of HSP70 or ML346. (*G*) IL-1β release in wildtype or HSP70 overexpressed (HSP70 OE) THP-1 cells in response to the stimulation with LPS and S100A8/A9. (*H*) The binding of HSP70 to NLRP3 were evaluated by co-immunoprecipitation analysis after LPS-primed THP-1 cells were treated with S100A8/A9 heterodimer. ∗*P* < .05, ∗∗*P* < .01, ∗∗∗*P* < .001, ns: not significant, *P* > .05. PI, propidium iodide.

Heat shock protein 70 (HSP70) is an evolutionarily conserved chaperone that safeguards lysosomal integrity by accelerating acid sphingomyelinase (ASM)-mediated hydrolysis of sphingomyelin, the accumulation of which otherwise precipitates lysosomal membrane permeabilization. We found that S100A8/A9 suppressed ASM activity, drove sphingomyelin accumulation, and triggered lysosomal membrane permeabilization, all of which were reversed when cells were pretreated with the HSP70 agonist ML346 or recombinant HSP70 (Figure 8*A–C*). Consistently, pharmacologic or exogenous HSP70 supplementation attenuated S100A8/A9-induced IL-1β release in LPS-primed THP-1 cells (Figure 8*D*) and mitigated AP-Exo–triggered pyroptosis and IL-1β secretion in MH-S cells (Figure 8*E* and *F*). Likewise, overexpression of HSP70 in THP-1 cells dampened IL-1β production in response to S100A8/A9 (Figure 8*G*). Given that HSP70 suppresses NLRP3 inflammasome signaling through direct binding and steric hindrance of NLRP3 assembly, we asked whether S100A8/A9 might promote inflammasome activation by disrupting this interaction. Coimmunoprecipitation assays revealed that S100A8/A9 treatment did not significantly affect HSP70–NLRP3 binding (Figure 8*H*), indicating that S100A8/A9 acts independently of HSP70 displacement. Instead, disruption of the HSP70–ASM axis emerges as a critical mechanism by which S100A8/A9 destabilizes lysosomes, licenses NLRP3 inflammasome activation, and drives pyroptotic cell death.

## Discussion

The progression of AP from a localized pancreatic inflammation to systemic inflammatory response, particularly the dissemination of inflammatory mediators to distant organs like the lungs, represents a critical event in the development of severe AP. Hence, it has been a conventional view that the inflammatory factors responsible for remote tissue injury in the process of AP progression originate primarily from the pancreas. However, cumulative evidence now indicates that once proinflammatory molecules in PAAF are absorbed and transported to the liver via the portal vein, the majority of these mediators are efficiently cleared by hepatic sinusoids. Consequently, the inflammatory mediators that ultimately enter the systemic circulation and trigger dysfunction in distant organs are unlikely to derive directly from the inflamed pancreas but are instead more plausibly generated as part of a secondary hepatic response. Bonjoch and colleagues previously demonstrated that the liver sequestered AP-associated exosomes,^6^ and we have further shown that the pulmonary accumulation of intraperitoneally injected AP exosomes was markedly lower than that achieved by intravenous delivery (Figure 5*A*). Collectively, the data support the view that circulating exosomes contributing to AP-related lung injury do not arise directly from the pancreas. Instead, pancreatic inflammatory signals are first filtered by the liver, which then generates and releases pathogenic exosomes into the systemic circulation as part of a secondary hepatic inflammatory response.

The liver-resident macrophages, KCs, have emerged as a critical intermediary linking primary pancreatic injury to extrapancreatic organ failure. To model this pathogenic relay, we exposed KCs to PAAFs, which are known to induce proinflammatory signaling, including NLRP3 activation and cytokine release, thereby recapitulating key features of hepatic macrophage activation during AP. Under these conditions, activated KCs released exosomes capable of inducing robust pyroptosis in AMs. Consistent with this mechanistic link, depletion of KCs prior to AP induction markedly reduced the pyroptotic potential of circulating exosomes both in vitro and in vivo, and concurrently alleviated lung injury. Although additional stimuli such as circulating cytokines may also contribute to exosome release in vivo, our model captures a physiologically relevant pathway of KC activation. Together, these findings define the liver as a major source of pathogenic exosomes during AP and highlight the pivotal role of KCs in shaping the intensity of systemic inflammation, offering a conceptual framework to explain the considerable heterogeneity in clinical trajectories observed among patients with AP.

Among the myriad molecular cargo of AP-derived exosomes, we identified the alarmin heterodimer S100A8/A9 as the principal instigator of AM pyroptosis and consequent lung injury. S100A8 and S100A9 are both calcium-binding proteins of the S100 family that are abundantly released by myeloid cells under stress. Although previous work has linked extracellular S100A8/A9 to NLRP3 inflammasome activation and pyroptosis,^12^^,^^13^^,^^21^ the underlying molecular mechanism remains fragmentary. Current models posit that the heterodimer engages pattern-recognition receptors, most notably TLR2/4 and RAGE, to trigger nuclear factor κB–dependent transcriptional priming of the NLRP3 machinery. Our findings clarify this paradigm by showing that S100A8/A9 elicited robust IL-1β release from LPS-primed THP-1 cells exclusively through TLR4, whereas pharmacologic blockade of TLR2 or RAGE produces no detectable impact on the response. Pharmacologic interrogation further demonstrated that both K^+^ efflux and cathepsin B activity are required for IL-1β release, as extracellular KCl, which prevents K^+^ efflux, and the cathepsin B inhibitor CA-074Me each abolished cytokine production. Remarkably, when S100A8/A9 was delivered via exosomes, it induced IL-1β secretion in a manner that no longer relied on TLR4 signaling or K^+^ efflux but remained strictly dependent on cathepsin B. This pattern suggests that fusion of the exosomal membrane with recipient cells may deliver S100A8/A9 directly into the lysosomal compartment, thereby bypassing the requirement for surface-receptor engagement and upstream ion-flux checkpoints. In this context, although exosome-associated S100A8/A9 appears to represent a more potent modality for eliciting inflammatory responses, the contribution of soluble S100A8/A9 should not be overlooked and merits further investigation. Collectively, these observations not only expand the functional repertoire of S100A8/A9 but also raise the possibility that AP exosomes harbor additional, as-yet-unidentified determinants of pyroptotic signaling.

Earlier work attributed NLRP3 inflammasome activation by S100A8 or S100A9 monomers to ROS generated through nicotinamide adenine dinucleotide phosphate oxidase.^21^^,^^22^ In contrast, the IL-1β response elicited by recombinant S100A8/A9 heterodimer or plasma exosomes was resistant to the ROS scavenger, NAC. These divergent results imply that the alarmin’s oligomeric state, either monomeric or heterodimeric form, likely determines the molecular pathway leading to NLRP3 inflammasome activation and the execution of pyroptosis. S100A8/A9 circulates mainly as a calcium-stabilized heterodimer, yet both monomeric and dimeric forms can drive distinct inflammatory programs, with S100A9 contributing the predominant signaling activity within the complex. It is not yet clear whether this functional hierarchy similarly dictates the pathway leading to pyroptosis. In present study, side-by-side comparison revealed that the heterodimer and S100A9 monomer were almost equipotent in driving pyroptosis of LPS-primed macrophages, whereas S100A8 monomer exhibited modestly reduced activity (Figure 7*G* and *H*). These data align with the reported rank order of proinflammatory potency among the 3 species and support therapeutic strategies that prioritize S100A9-directed neutralizing antibodies or small-molecule antagonists to prevent AP aggravation. Notably, although S100A9 inhibition or deletion markedly attenuated AP-Exo–induced pyroptosis and lung injury, the effects were not completely abolished. This residual activity may reflect the presence of S100A8 monomers, which were not fully neutralized by S100A9-targeted approaches but can also induce NLRP3 activation. Therefore, the residual propyroptotic effects observed could be partially attributed to exosomal S100A8 monomers, highlighting the potential contribution of multiple S100A8/A9 forms in mediating AP-Exo–induced lung injury.

Lysosomal disruption with subsequent cathepsin B escape is broadly recognized as a key initiating signal for NLRP3 inflammasome activation. Interventions that preserve lysosomal stability and restrain cathepsin B activity have therefore proved effective in curbing NLRP3-dependent pyroptosis. The evolutionarily conserved chaperone HSP70 fulfils this cytoprotective function by docking to bis(monoacylglycero)phosphate, an interaction that enhances ASM activity and averts sphingomyelin-driven destabilization of the lysosomal membrane.^23^, ^24^, ^25^ Moreover, HSP70 was previously shown to suppress NLRP3 signaling by binding directly to the sensor protein, thereby sterically hindering inflammasome assembly.^26^ In addition, the chaperone sequestered cathepsin B, reducing its access to NLRP3 and further restraining inflammasome activation.^27^ Our data do not support a mechanism in which S100A8/A9 relieves NLRP3 from HSP70-dependent suppression (Figure 8*H*). Instead, we found that the alarmin blunted ASM activity, leading to sphingomyelin accumulation, lysosomal membrane rupture, and cathepsin B release, a sequence that culminated in NLRP3 inflammasome activation (Figure 8*A–C*). The ensuing surge in cathepsin B activity may also exceed the capacity of HSP70 to sequester the protease, thereby weakening its ability to restrain inflammasome engagement. Therefore, our findings unveil a previously unrecognized mechanism by which S100A8/A9 licenses NLRP3 activation through lysosomal destabilization and highlight that this effect is tightly linked to suppression of the HSP70–ASM axis. Beyond its direct engagement with NLRP3, HSP70 also restrains inflammasome activation by maintaining ASM-dependent lysosomal membrane stability, thereby refining our understanding of how this chaperone sets the threshold for NLRP3 signaling.

Notwithstanding these insights, several limitations of the present study should be acknowledged. First, our conclusions are primarily derived from in vitro and experimental models and have not yet been validated in clinical samples, which will be essential to establish their translational relevance. Second, our investigation focused predominantly on AMs, whereas the potential contributions of other pulmonary cell populations, such as alveolar epithelial cells and endothelial cells, remain to be elucidated. Given the complex cellular interplay underlying lung injury, clarifying the roles of these additional cell types will be important for a more comprehensive understanding of the pathogenic process. Third, because dynamic monitoring of circulating exosomal S100A8/A9 levels was not performed, the relationship between their temporal fluctuations and the time window of AP-associated lung injury remains undefined. Addressing these issues in future studies will help to refine the mechanistic framework and further substantiate the pathophysiological significance of our findings.

## Conclusions

Taken together, these findings delineate a previously unrecognized pancreas–liver–lung axis in which Kupffer cell–derived exosomes act as the principal mediators of AP-associated lung injury. Exosomal S100A8/A9 suppresses HSP70-mediated ASM activity, driving sphingomyelin accumulation, lysosomal destabilization, and subsequent NLRP3-dependent pyroptosis in AMs. By positioning exosomal S100A8/A9 as a potential mechanistic driver and a candidate therapeutically accessible target, this work provides a conceptual framework that may inform the development of precision interventions for AP and its life-threatening extrapancreatic sequelae.

## Materials and Methods

### Materials

Compounds including MCC950, Paquinimod, TAK-242, C29, FPS-ZM1, ML346 and CA-074Me were purchased from MedChemExpress. NAC was obtained from Solarbio Science and Technology Co Ltd. LPS was from *Escherichia coli O111:B4*; L-Arg and TC were from Sigma Aldrich. Nigericin was purchased from Invitrogen. The clodronate liposomes were obtained from Liposoma BV. Antibodies against GSDMD and S100A9 were obtained from Abcam. Lysotracker Red DND-99 and BODIPY-FL C12-Sphingomyelin were purchased from Thermo Fisher Scientific. Recombinant human S100A8/A9 heterodimer protein and ELISA kits for mouse S100A8/A9, tumor necrosis factor α, IL-6, and mouse/human IL-1β were obtained from R&D Systems.

### Experimental Model of Acute Pancreatitis and Treatments

Male C57BL/6 mice were obtained from Chengdu Dossy Experimental Animals Co, Ltd. S100A9 KO mice were generated by Cyagen Biosciences Inc. The L-Arg–induced AP model was created by intraperitoneal injections of L-Arg solution at 2 doses of 4 g/kg body weight each 1 hour apart. A necrotizing AP model was established using a retrograde injection of 50 μL of saline containing 5% TC into the pancreatic duct. Animals were sacrificed by CO_2_ asphyxiation 24 hours after AP induction followed by immediate collection of blood and tissue samples. For preparation of PAAF, 6 hours after the induction of TC-induced AP in mice, the peritoneal exudate was aseptically collected from the abdominal cavity followed by a centrifugation at 2500 × g for 15 minutes at 4 °C. The supernatant was passed through a 0.2-μm filter and collected as PAAF. Bacterial contamination was checked by culturing PAAF in Luria-Bertani broth for 48 hours. All contaminated samples were discarded. The amount of endotoxins in PAAF was detected using a Chromogenic Limulus Amoebocyte Lysate Endotoxin Assay Kit (Beyotime). The result showed a concentration of less than 0.1 EU/mL of endotoxin in the PAAF samples, excluding the possibility of endotoxin contamination. The animal care and experimental protocols were approved by the Institutional Animal Care and Use Committee of University of Electronic Science and Technology of China, in accordance with National Institutes of Health guidelines for the Care and Use of Laboratory Animals.

### Collection of Bronchoalveolar Lavage Fluid

After animals were sacrificed, bronchoalveolar lavage (BAL) was immediately carried out by flushing the lungs with 0.5 mL 37 °C sterile physiological saline and withdrawing the fluid for 3 times via the tracheal cannula. The BALF was pooled together followed by a centrifugation at 1500 × g for 5 minutes at 4 °C. The supernatant was stored at −80°C for subsequent quantitation of cytokine levels by ELISA. For total and differential cell counting, the cell pellets were resuspended in 1 mL of phosphate-buffered saline (PBS). The total cell count was determined with a Neubauer chamber, and differential cells counts were carried out (at least 300 cells) using cytospin preparation stained with Diff-Quick (Solarbio).

### Evaluation of Pulmonary Vascular Permeability

Pulmonary vascular permeability was assessed by Evans blue dye extravasation as described previously.^10^ Briefly, mice were injected via the tail vein with Evans blue dye (20 mg/kg) 30 minutes before the animals were sacrificed. BAL was performed, and then the lungs were perfused, removed, and immersed in formamide at room temperature for 24 hours for the extraction of the dye. The absorbance of Evans blue dye extracted in formamide was measured at a wavelength of 620 nm.

### Histopathologic Evaluation and Immunofluorescence Assay

Collected lung tissue samples were fixed in formaldehyde and subsequently embedded in paraffin. Then samples were sectioned and stained with hematoxylin and eosin, using a standard staining procedure. Histopathologic evaluation was performed under light microscope by an experienced laboratory pathologist who was blinded to the group identity for the samples in accordance with the criteria reported by Su et al.^28^ Activation of caspase-1 in AMs during AP was determined by dual immunofluorescent staining with anti-F4/80 and anti–caspase-1 monoclonal antibodies. Briefly, formaldehyde-fixed lung sections were dewaxed in xylene and rehydrated in ethanol/water, followed by an incubation with sodium citrate repair solution. After being treated with 3% hydrogen peroxide to quench the endogenous peroxidase, the sections were incubated with 1% bovine serum albumin to block nonspecific sites. Double immunofluorescence staining was performed using primary antibodies for caspase-1 (Santa Cruz Biotechnology) and F4/80 (Cell Signaling Technology). The secondary antibodies used were fluorescein isothiocyanate–labeled goat anti-mouse IgG for caspase-1, and Cy3-labeled goat anti-rabbit IgG for F4/80 (Servicebio). The 4', 6-diamidino-2-phenylindole (DAPI) was used as a nuclear counterstain. Finally, the images were observed under a confocal microscope (Zeiss).

### Isolation of Alveolar Macrophages in Bronchoalveolar Lavage Fluid

The collected BALF was centrifuged at 600 × g for 10 minutes at 4 °C. Then the cell pellets were resuspended in the complete culture medium (Dulbecco’s Modified Eagle Medium [DMEM] with 10% fetal bovine serum [FBS]) and were subsequently seeded in 96-well plates at the density of 1 × 10^5^ cells/well at 37 °C in 5% CO_2_ incubator for cell adherence. After incubation, the nonadherent cells were washed away with sterile PBS 2 times, and adherent macrophages were further incubated in fresh culture medium overnight to make them quiescent. The viability of the cells was determined by trypan blue exclusion test.

### Isolation of Exosomes

Exosomes were isolated from mice plasma using the Total Exosome Isolation Reagent (Invitrogen) according to the manufacturer’s instructions. In brief, plasma samples were centrifuged at 2000 × g for 30 minutes to remove any cells and debris. Then 0.5 volumes of the Total Exosome Isolation Reagent were added to the cell-free media followed by an incubation at 4 °C overnight. After centrifugation at 10,000 × g for 1 hour at 4 °C, the supernatant was discarded. Then, the centrifugation and aspiration steps were repeated. Finally, the pellets containing the exosomes were resuspended in PBS. The particle size and the concentration of the exosomes were measured using a Zetasizer Nano ZS system (Malvern Instruments). The expression of exosomal S100A9 and surface marker TSG101 were identified by Western blotting. S100A8/A9 level in exosome was quantified by ELISA. For transmission electron microscopy analysis, the isolated exosomes were loaded on copper grids and dried for 20 minutes. After twice washing with PBS, the sample was negatively stained by phosphotungstic acid for 10 minutes. Then images were taken by a JEM-2100 microscope (JEOL).

To observe the exosome uptake for AMs, exosomes were stained by PKH26 Red Fluorescent Cell Linker Dye (Sigma Aldrich). The staining reaction was stopped with 3% bovine serum albumin for 1 minute, and labeled exosomes were washed with PBS 3 times to remove the unbound dye. Primary AMs or the immortalized murine AM, MH-S cells, were cultured with 200 μL PBS containing 20 μg PKH26-stained exosomes for 2 hours at 37 °C. Then cells were washed 3 times with PBS and fixed with 4% paraformaldehyde. After DAPI staining, images were captured by a confocal microscope. Furthermore, to observe the exosome uptake in vivo, PKH26-labeled exosomes (PKH26-Exo) at a dose of 2 μg of exosome protein, were administrated through intraperitoneal or tail vein injection to mice. Twenty minutes after administration, mice were sacrificed, and the lungs were harvested for fluorescence analysis with a confocal microscope. In another experiment, the role of AP-conditioned exosomes in delivering S100A9 to AMs was tested by incubating primary AMs isolated from S100A9 KO mice with the exosomes. Cells were cultured overnight in a 24-well plate with complete culture medium. After being washed with serum-free medium, AMs were treated with exosomes (20 μg per well) at 37 °C for 2 hours. Cellular S100A9 was probed by rabbit primary antibody and fluorescein isothiocyanate–labeled secondary antibody and visualized under a confocal microscope. Additionally, for the depletion of exosomes from plasma, plasma samples were mixed with a 50% solution of PEG (MW 10,000) at a sample-to-PEG ratio of 5:1. The mixture was incubated at 4 °C for 2 hours and subsequently centrifuged at 1500 × g for 30 minutes at 4 °C. The final supernatant was collected and designated as exosome-depleted plasma.

### Cell Culture and Transfection

The human THP-1 cells purchased from American Type Culture Collection were differentiated with phorbol myristate acetate and cultured with RPMI 1640 medium containing 10% FBS at 37 °C with 5% CO_2_. The lentivirus-mediated HSP70 overexpression vector was obtained from GeneChem. The THP-1–derived macrophages were transfected with the packaged virus particles (multiplicity of infection = 100) and polybrene for 24 hours. Afterwards, the medium was replaced by RPMI 1640 medium containing 10% FBS, and the cells were further incubated for 3 days. Western blot analysis was used to assess the infection efficiency before subsequent experiments.

### Determination of Pyroptosis

To evaluate the ability of exosomes isolated from plasma of AP mice to induce AM pyroptosis, primary AMs or MH-S cells were cultured overnight in a 24-well plate at a density of 1 × 10^5^ cells per well with complete culture medium. After being washed with serum-free medium, cells were incubated with exosomes for 24 hours. Pyroptosis was evaluated by measuring the level of activated caspase-1, release of LDH, production of IL-1β, and protein expression of N-terminal GSDMD fragment (GSDMD-NT). The caspase-1 activity was quantified using FAM-FLICA Caspase-1 Assay Kit (Immunochemistry Technologies) according to the manufacturer's instructions. Briefly, cells were incubated with medium containing carboxyfluorescein-labeled fluorescent-labeled inhibitor of caspase (FLICA) for 1 hour and subsequently washed 3 times to diffuse unbound FLICA. Nuclei were stained by incubation with DAPI for 5 minutes. Cells were cultured in 12-well plates, and activated caspase-1 was imaged using a confocal microscope. LDH activity in the cell culture supernatant was detected by LDH Cytotoxicity Assay Kits (Biovision). In brief, the supernatants were transferred into 96-well plates and mixed with LDH reaction solution. After an incubation of 30 minutes at room temperature, the absorbance was quantified at 450 nm using a microplate spectrophotometer (Thermo Fisher Scientific). As a control, cells were lysed with 1% Triton X-100 to determine maximal LDH release (100%). The levels of IL-1β in supernatants were analyzed by ELISA. Protein expression of GSDMD-NT was detected by immunoblotting after protein extraction and quantitation.

Pyroptosis in phorbol myristate acetate–differentiated THP-1 cells was elicited when LPS-primed cells were stimulated with nigericin (7.25 μg/mL) or S100A8/A9 (0.5 μg/mL). One hour later, pyroptosis was evaluated by measuring the level of activated caspase-1, release of LDH, production of IL-1β, and protein expression of GSDMD-NT as described above. For the evaluation of AMs pyroptosis in vivo, collected AMs from BALF were stained with both FAM-FLICA and propidium iodide (Immunochemistry Technologies) and then analyzed by flow cytometry.

### Kupffer Cells Isolation and Treatment

To isolate KCs from liver tissue, the mouse liver was perfused via the inferior vena cava with pre-warmed (37 °C) Hank’s buffered salt solution (Ca^2+^ and Mg^2+^ free) until there was no evidence of blood in the spent perfusion medium. Then the liver was perfused with prewarmed dissociation buffer (0.4% type I collagenase in Hank’s buffered salt solution) with for 5 minutes. Perfused livers were dissected and filtered through a sterile 100-μm nylon cell strainer to obtain single-cell suspensions. Hepatocytes were removed from cell suspensions after a centrifugation at 50 × g for 3 minutes at 4 °C. The supernatant was centrifuged at 400 × g for 7 minutes, and the pellet was suspended in 15% Opti-Prep (Sigma Aldrich) followed by a centrifugation at 1400 × g for 21 minutes at 4 °C. Cells were collected from the interface and then seeded into 6-well plate at a density of 1 × 10^7^ per well in DMEM and incubated for 2 hours at 37 °C. After washing with PBS, nonadherent cells were then removed, and the adherent cells were KCs.

To obtain KC-derived exosomes, KCs were treated with PAAF (5% diluted in DMEM medium) at 37 °C for 6 hour. The culture medium of KCs was replaced by a medium containing 10% exosome-depleted FBS before the exosomes were isolated from the cell supernatant. Exosomes were isolated following the procedure as described above.

Depletion of KCs in vivo was performed as described previously.^29^ Briefly, 24 hours prior to AP induction, each mouse was intravenously injected with 200 μL of clodronate liposomes suspension (1 mg/mL) to selectively deplete KCs.

### Analysis of Lysosomal Integrity

LysoTracker Red DND-99, a fluorescent dye that stains acidic intracellular components such as lysosomes in living cells, was used to investigate lysosomal rupture in response to S100A8/A9. After THP-1 cells were harvested and washed with PBS, the cells were resuspended in prewarmed medium containing 100 nM DND-99 and then incubated for 90 minutes at 37 °C in the dark. The fluorescence intensity was analyzed using a flow cytometry analyzer (BD Biosciences) after cells were washed to remove the unbound dye.

Cathepsin B activity was measured using the Assay Kits purchased from Abcam according to the manufacturer’s instructions. In brief, cells were harvested and lysed in chilled lysis buffer. After an incubation on ice for 10 minutes, lysate samples were centrifuged at 15,000 × g for 5 minutes. Then the supernatant was transferred to black 96-well plates and incubated with the substrate buffer solution at 37 °C for 1 hour in the dark. The fluorescence from the cathepsin B cleaved substrate was measured using a fluorescent microplate reader at excitation/emission of 400/505 nm. Cathepsin B activity was represented as the fold increase in the fluorescence intensity compared with that of control.

### Evaluation of Acid Sphingomyelinase Activity

THP-1 cells were lysed by performing multiple freeze–thaw cycles with liquid nitrogen. Then the lysate was centrifuged at 10,000 × g for 10 minutes at 4 °C, and the supernatant was collected for determining the protein content employing the bicinchoninic acid protein assay kit (Sangon). The intracellular activity of ASM was measured using the Acid Sphingomyelinase Assay Kit (Abcam) in accordance with the manufacturer’s recommendations. Briefly, samples were added to a 96-well plate and incubated with an ASM-specific fluorogenic substrate for 3 hours at 37 °C, protected from light with shaking. Afterwards, the fluorescence signal (excitation/emission = 540/590 nm) was detected by a microplate reader. The ASM activity was represented as relative activity normalized to the control sample. The ASM activity was also determined using BODIPY FL C_12_-Sphingomyelin, a fluorescent substrate analogue for ASM that is often used to stain cells for tracking sphingomyelin accumulation due to ASM activity inhibition. Briefly, BODIPY FL C_12_-Sphingomyelin was added to the cells followed by an overnight incubation at 37 °C to incorporate into cell membranes. The cells were then fixed in 4% paraformaldehyde solution containing 1 μg/mL DAPI for 30 minutes at room temperature in the dark, followed by 3 washes with PBS. The fluorescence images were captured by a laser confocal microscope.

### Statistical Analysis

GraphPad Prism software was used for data visualization and analysis. All experimental data are expressed as mean ± standard deviation. Pearson correlation coefficient was calculated to assess the correlation. Statistical comparisons between groups were examined by Student’s *t* test or 1-way analysis of variance. Differences in values were considered significant if *P* < .05. The statistical significance between 2 levels is represented by asterisks (∗ .01 < *P* < .05; ∗∗ .001 < *P* < .01; ∗∗∗ .0001 < *P* < .001).

## Supplementary Material

Supplementary Material

Extended PDF
