# Supplementary figures and images for: Kupffer Cell–Derived Exosomal S100A8/A9 Promotes Inflammasome–Dependent Pyroptotic Lung Injury in Acute Pancreatitis
Source: Cell Mol Gastroenterol Hepatol. 2026 May 12;20(9):101810. doi: 10.1016/j.jcmgh.2026.101810 (PMC13311184; doi:10.1016/j.jcmgh.2026.101810)

Fig. 2C

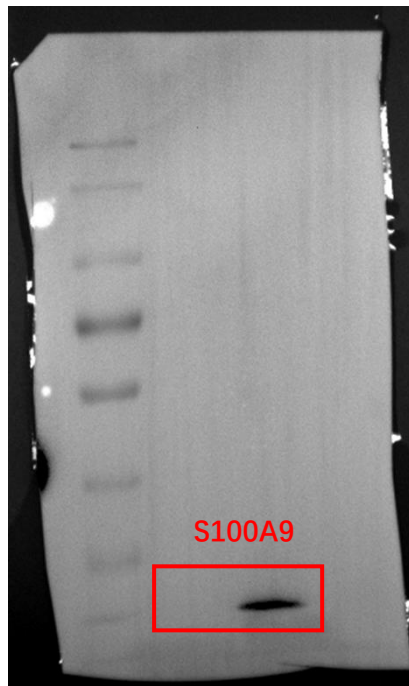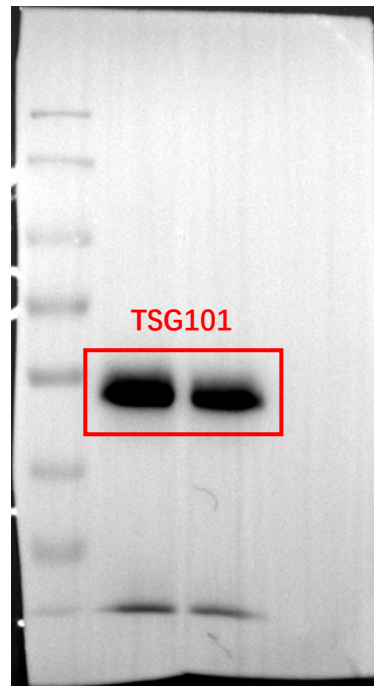

Fig. 3B

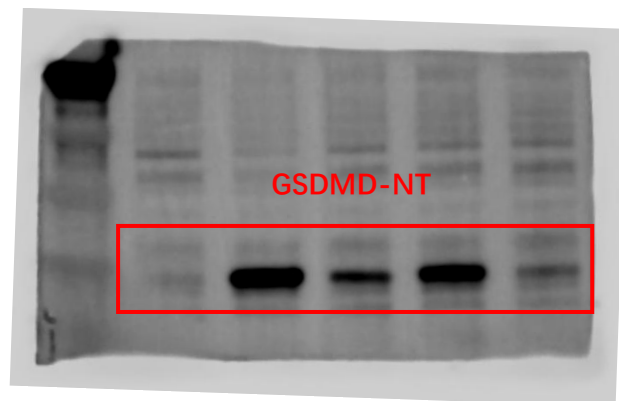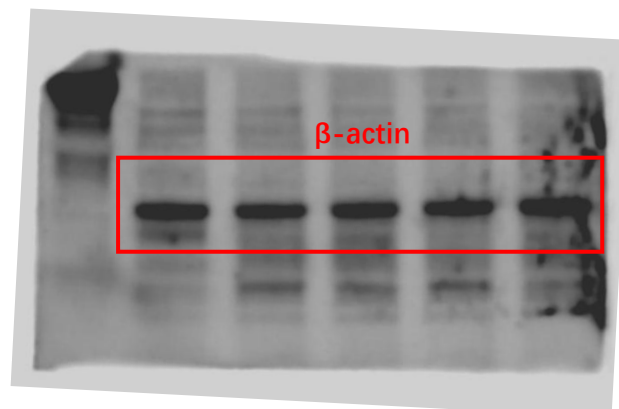

Fig. 3H

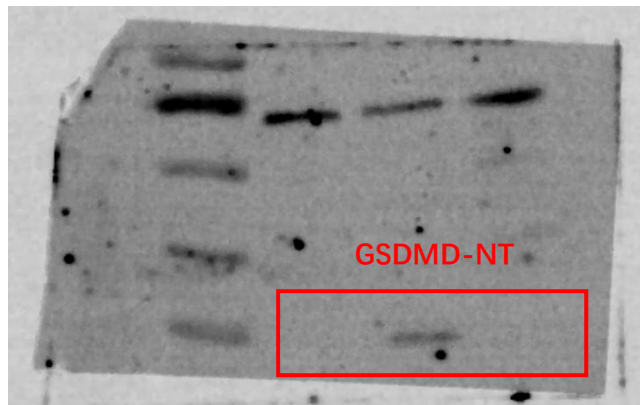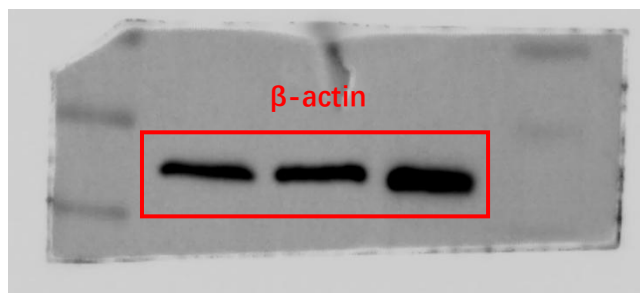

Fig. 7D

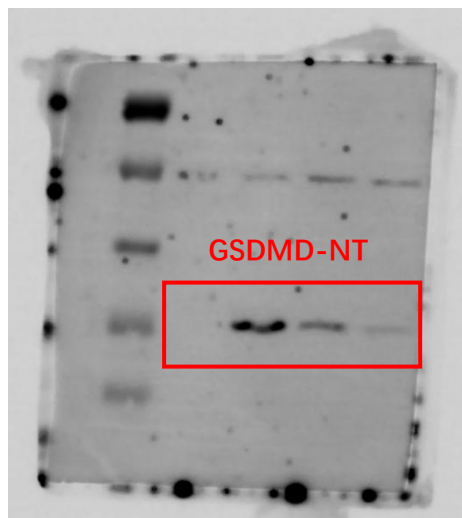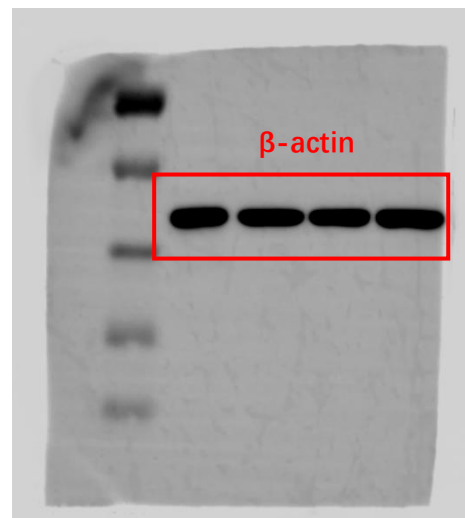

Fig. 8H

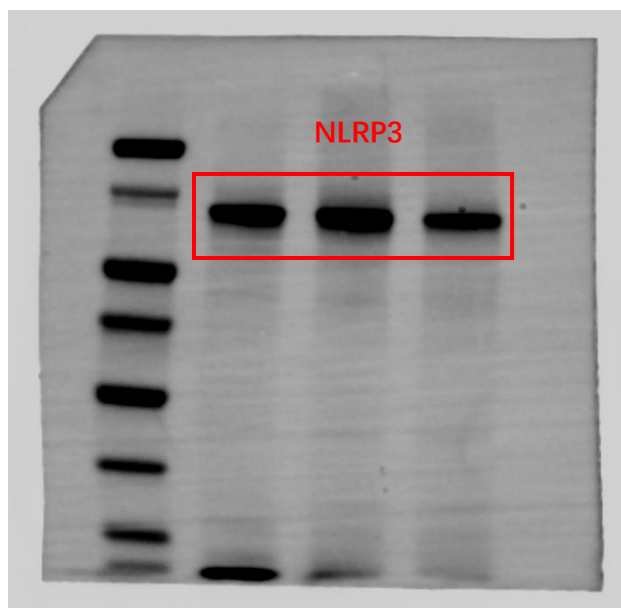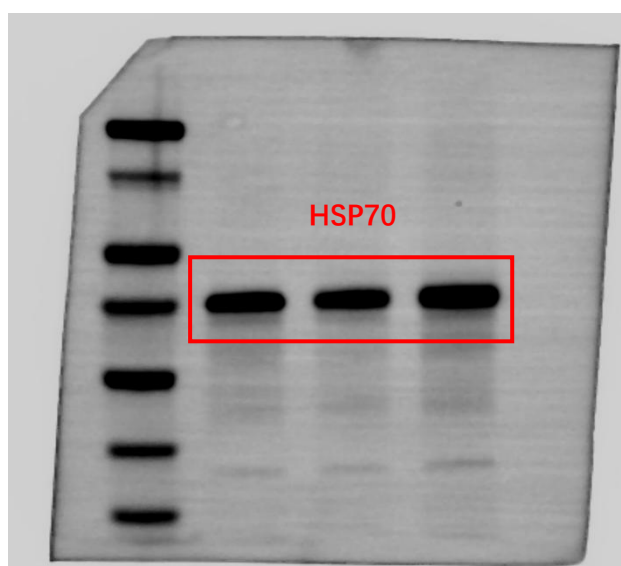

Supplement: Supplementary Material [file mmc1.pdf]
